# Understanding Solid–Gas Reaction Mechanisms by Operando Soft X-Ray Absorption Spectroscopy at Ambient Pressure

**DOI:** 10.1021/acs.jpcc.0c02546

**Published:** 2020-06-05

**Authors:** Luca Braglia, Martina Fracchia, Paolo Ghigna, Alessandro Minguzzi, Daniela Meroni, Raju Edla, Matthias Vandichel, Elisabet Ahlberg, Giuseppina Cerrato, Piero Torelli

**Affiliations:** † CNR- Istituto Officina dei Materiali, TASC, 34149 Trieste, Italia; ‡ Dipartimento di Chimica, Università di Pavia, V.le Taramelli 13, I-27100 Pavia, Italy; § Dipartimento di Chimica, Università degli Studi di Milano, Via Golgi 19, 20133 Milan, Italy; ∥ INSTM, Consorzio Interuniversitario per la Scienza e Tecnologia dei Materiali, Via Giusti 9, 50121 Firenze, Italy; ⊥ Department of Chemical Sciences and Bernal Institute, Limerick University, V94 T9PX Limerick, Ireland; # Department of Chemistry and Molecular Biology, University of Gothenburg, Kemigården 4, SE-412 96 Gothenburg, Sweden; ∇ Department of Chemistry and NIST Interdipartimental Center, Università degli Studi di Torino, via P. Giuria, 7, 10125 Torino Italy

## Abstract

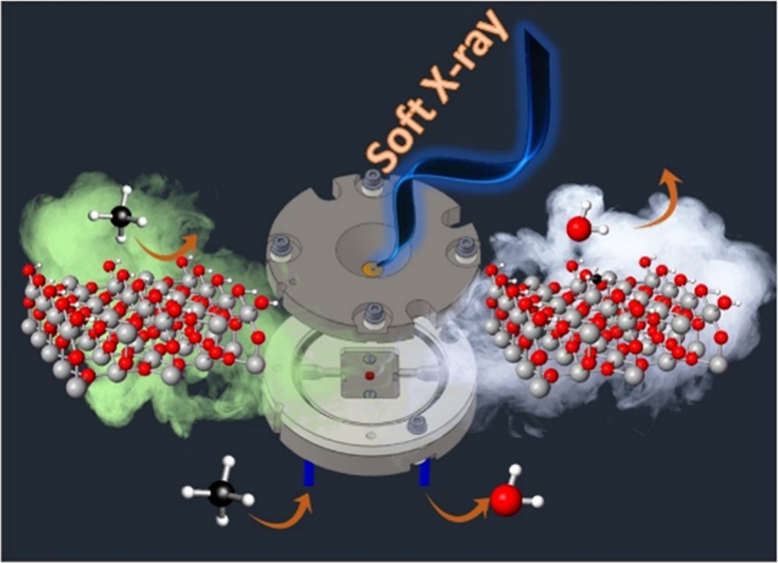

Ambient-pressure operando soft X-ray absorption spectroscopy (soft-XAS) was applied to study the reactivity of hydroxylated SnO_2_ nanoparticles toward reducing gases. H_2_ was first used as a test case, showing that the gas phase and surface states can be simultaneously probed: Soft-XAS at the O K-edge gains sensitivity toward the gas phase, while at the Sn M_4,5_-edges, tin surface states are explicitly probed. Results obtained by flowing hydrocarbons (CH_4_ and CH_3_CHCH_2_) unequivocally show that these gases react with surface hydroxyl groups to produce water without producing carbon oxides and release electrons that localize on Sn to eventually form SnO. The partially reduced SnO_2 – *x*_ layer at the surface of SnO_2_ is readily reoxidized to SnO_2_ by treating the sample with O_2_ at mild temperatures (>200 °C), revealing the nature of “electron sponge” of tin oxide. The experiments, combined with DFT calculations, allowed devising of a mechanism for dissociative hydrocarbon adsorption on SnO_2_, involving direct reduction of Sn sites at the surface via cleavage of C–H bonds and the formation of methoxy- and/or methyl-tin species at the surface.

## Introduction

Understanding the mechanism of solid/gas chemical reactions is crucial in many processes, either at low (e.g., fuel cells) and at mid-high temperatures (hydrocarbon chemistry, sensors, etc.). A major example is the mechanism of high-temperature gas sensing on semiconducting materials, where the interaction of the analyte with the solid surface is the key factor for the correct behavior of the sensor; however, the detailed mechanism of this interaction is still unclear. Similarly, hydrocarbon oxidation (C–H activation) on metal oxide catalysts is a rather unknown process, yet of paramount importance in several fields.

Semiconducting oxides such as SnO_2_ act as gas sensors as their surface conductivity changes in response to variation of the surrounding atmosphere.^1−3^ The adsorption of gas molecules supplies donor or acceptor levels in dependence on the reductive or oxidative nature of the analyte compared to molecular oxygen. While a number of comprehensive review articles and books dealing with the mechanisms of semiconducting oxides and the various aspects involved in gas-sensing processes have been published,^2,4−6^ a general consensus toward a unified model describing their functioning is still lacking.^7−13^ SnO_2_ has also a wide range of applications as a catalyst for oxidation reactions, for example, the CO/O_2_ and CO/NO reactions, suggesting that the ease of oxidation/reduction of surface states plays a major role in the reaction mechanisms.^14^

A complete understanding of the reaction mechanism, including the generation of byproducts in both phases and/or at the interface is required in order to design tailored materials. As it happens in the study of any heterogeneous phenomenon, conventional investigation methodologies give either unclear or incomplete information. The main reason is that typical probes focus on a single “actor” of the reaction (i.e., catalyst surface, adsorbed intermediates, and gas phase). In the best case, as for the so-called hyphenated techniques, two or more probes are used to monitor different portions of the interface. However, no single operando (i.e., under operative conditions) technique is capable to simultaneously look at highly complex processes from several perspectives, considering all parts of the interface (gas, solid surface, and adsorbed intermediates) and the relevant events occurring in mutual correlation.

A correlated problem is the real nature of the surface. Water species are ubiquitous on oxide surfaces, and these species are then expected to play a role in the surface reactivity. For example, it has been demonstrated that CO_2_ cannot be adsorbed on heat-treated SnO_2_ in dry air.^15^ As the relative humidity increases, CO_2_ is adsorbed, forming carbonate on the surface. When the surface is fully hydroxylated, carbonate is adsorbed by replacing surface −OH groups.^15^ However, the presence of hydroxyl groups is scarcely taken into account when dealing with the mechanisms of adsorption on SnO_2_: This is mainly due to the lack of established tools for looking at oxide surfaces in real operando conditions.

A wide collection of invaluable probes in material science is provided by synchrotron-based hard X-ray techniques: Hard X-ray diffraction and spectroscopies are presently the working horses of material characterization, both ex and in situ. For example, hard X-ray absorption spectroscopy (XAS) is an irreplaceable tool in the investigation of local atomic and electronic structures of materials.^16,17^ Moreover, operando XAS experiments with hard X-rays are well-established, and almost every experimental condition can be reached to simulate realistic reaction environments.^18−20^ On the contrary, in the soft X-ray regime, the applications of XAS (soft-XAS) in material science were substantially limited to a “surface science” approach, i.e., to the study of clean surfaces in high-vacuum conditions. In fact, the low penetration depth of X-rays with energies lower than 1 keV and the severe vacuum limitation have somehow hindered the development of operando experiments. However, soft-XAS is expected to give invaluable information for a complete understanding of the mechanisms of phenomena taking place at material surfaces and interfaces, such as catalysis, intercalation, electrochemistry, etc. Until now, these subjects have been mainly the playground of X-ray photoelectron spectroscopy (XPS), which is indeed sensitive to the immediate surface. However, despite some latest developments,^21^ even in the near-ambient-pressure variant (NAP-XPS), the experiments are conducted in the mbar or tens of mbar range, which is very far from real working conditions. In this sense, soft-XAS was recently pushed forward in order to overcome this so-called “pressure gap”.^22^ For instance, transmission mode and fluorescence mode cells for soft-XAS have been developed, allowing operando investigations, for example, in the field of electrochemistry;^23−25^ only very recently, specific reaction cells were designed for the study of catalyst or electrode surfaces at high temperature and at atmospheric pressure.^26−32^ In these cases, soft-XAS is operated in the total electron yield (TEY) detection method, which renders the technique intrinsically surface-sensitive, and allows effective probing of the pertinent surface states. Indeed, in the TEY mode, all electrons (photoelectrons, Auger electrons, and secondary electrons) emitted from the sample after photoabsorption are collected. Since the escape depth of such electrons at moderate kinetic energies is very small (of the order of few nanometers), only very few atomic layers below the surface can be probed.

In this work, we further push forward soft-XAS operando experiments by demonstrating that the surface states and the reaction products at a solid/gas interface in solid/gas heterogeneous process can be probed simultaneously at ambient pressure and in a wide range of temperatures. It is clear from all of the above that soft-XAS in situ and/or operando conditions will allow us to obtain fundamental information in this case. If reactions taking place on oxide surfaces are considered, oxygen states are expected to be deeply involved; in addition, gas-phase water can be formed through reaction(s) involving surface hydroxyl groups. All these oxygen species can be easily detected at the O K-edge at 543.1 eV, i.e., well within the soft X-ray regime. Furthermore, the M_4,5_-edges of tin at ca. 485–495 eV are close in energy and can be probed in the same experiment, thus permitting access of all valence states of the materials, searching for variations in the oxidation state of Sn.^33,34^

We performed experiments at the O K- and Sn M_4,5_-edges at ambient pressure with the aim of studying the mechanisms of the surface reaction with reducing (H_2_, CH_4_, and CH_2_CHCH_3_) gases by SnO_2_ nanoparticles. The investigation was conducted in the 100–360 °C temperature range, which nicely fits the operative range of the nanoparticles as a gas sensor.^35^ In these conditions, the SnO_2_ nanoparticles are free of physisorbed water, but chemisorbed, hydroxylated species are still present. H_2_ was chosen as a test gas to assess the capability of XAS to simultaneously detect water in the gas phase at the O K-edge and reduced Sn species in the nanoparticles at the Sn M_4,5_-edges. For what concerns CH_4_ and CH_2_CHCH_3_, the results unequivocally show that hydrocarbons react with surface-hydroxylated species and release electrons that localize on Sn states to eventually form a SnO layer. This mechanism for the reactive adsorption of hydrocarbons on SnO_2_ was finally tested against DFT calculations.

## Experimental and Theoretical Methods

### SnO_2_ Synthesis and Characterization

Nanocrystalline SnO_2_ was prepared by precipitation from aqueous solution of SnCl_4_, obtained by dissolving 0.01 mol of SnCl_4_·5H_2_O in 90 mL of MilliQ. Then, 210 mL of a 0.33 M urea aqueous solution was added. All reagents were purchased at analytical grade from Sigma-Aldrich and were used without further purification. The reaction mixture was stirred for 8 h at 90 °C and then overnight at room temperature. The resulting precipitate was washed by centrifugation–resuspension cycles with water/ethanol. It was then suspended in water and treated in a Teflon-lined autoclave for 4 h at 130 °C. The powder was then dried in an oven at 80 °C and calcined for 6 h at 400 °C under O_2_ flux.

The X-ray powder diffraction (XRPD) pattern was recorded using a Bruker D8 Advance diffractometer in Bragg–Brentano configuration, equipped with a Cu anticathode (Cu Kα, λ ≈ 1.54 Å). The sample specific surface area was determined from N_2_ adsorption–desorption isotherms in subcritical conditions (Coulter SA 3100) according to the Brunauer–Emmett–Teller (BET) method. Fourier transform infrared (FTIR) spectra were recorded on the as-synthesized sample using a Perkin Elmer Spectrum 100 spectrophotometer working in attenuated total reflectance (ATR) mode. Spectra were acquired using a resolution of 4.0 cm^–1^ and a total of 12 scans between 4000 and 400 cm^–1^.

Thermogravimetric analysis (TGA) curves were acquired on a Mettler-Toledo TGA/DSC 3+ STAR System in the 30–900 °C range with a 5 °C min^–1^ heating rate under N_2_ flux.

High-resolution transmission electron microscopy (HRTEM) images were obtained on a JEOL 3010–UHR instrument equipped with a LaB_6_ filament (acceleration potential, 300 kV). All digital micrographs were acquired by an Ultrascan 1000 camera, and image processing was performed by a Gatan Digital Micrograph program version 3.11.1. Samples were dry dispersed onto Cu grids coated with a “lacey” carbon film before analysis.

Raman spectra were recorded on the plain powder by means of a Bruker Vertex 70 spectrometer, equipped with the RAMII accessory and Ge detector, by exciting samples with a Nd:YAG laser source (1064 nm). Spectral resolution was 4 cm^–1^.

### XAS Experiment

**Figure 1:**
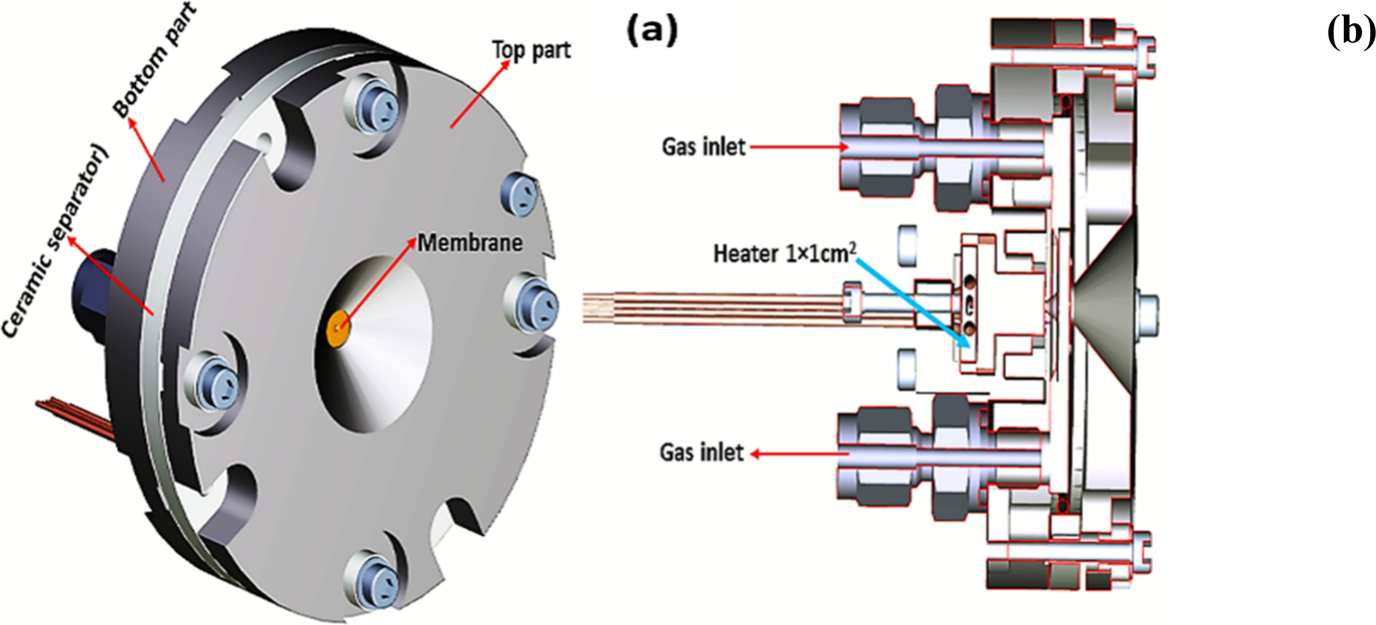
(a) 3D drawing of the front part of the reactor cell for ambient-pressure operando XAS experiments, as seen from the X-ray beam. The reactor cell contains a gas fluxing circuit and a Si_3_N_4_ membrane (window), of 100 nm thickness. The membrane separates the sample lodging, which is at ambient (1 atm) pressure, from the rest of the beamline, which is in high-vacuum conditions, permitting the transmission of the incident soft X-ray beam without significant loss of intensity in the photon energy range of interest. (b) Vertical section of the reactor cell in which the gas circuit, the heater, and the sample environment are clearly visible.

To obtain high-quality in situ soft-XAS data, the reaction cell shown in Figure 1 was used.^36^ The cell is installed at the advanced photoelectric effect high-energy (APE-HE) beamline at the ELETTRA synchrotron radiation source and can operate in the temperature range from RT to ca. 360 °C. The XAS detection in total electron yield (TEY) mode was achieved by measuring the drain current from the sample with a picoammeter. The monochromator of the APE-HE beamline allows continuous scanning of the X-ray energies; this, combined with the high signal-to-noise ratio of TEY, makes it possible to record XAS spectra in very short times, of the order of few seconds. For the XAS experiment, a small amount of the SnO_2_ nanoparticles (5 mg ca.), in the form of loose powder, was hand-pressed on the sample holder of the reaction cell of the APE beamline at the ELETTRA synchrotron radiation facility. The sample holder is fixed with screws onto the titanium base of the cell, which is floating from the ground and connected with a coaxial cable. In this geometry, the X-ray beam passes through the membrane and the gas layer then hits the sample and generates the secondary emission, which is collected by a picoammeter connected to the sample and measuring the drain current. All measurements were performed by keeping the sample grounded through the picoammeter and applying a positive bias voltage of 40 V to the membrane. The cell is mounted in the ultrahigh-vacuum chamber of the APE-HE beamline, coaxially with the X-ray beam. The reaction cell was mounted on an *x*-*y* table that allows its movement in the plane perpendicular to the incident beam with 5 μm vectorial precision. This allows the alignment of the membrane onto the beam. The sample surface, inside the cell, sits on the focal point of the beamline. The measurements were performed at the O K-edge, between 528 and 547 eV, and at the Sn M_4,5_-edges, between 480 and 510 eV, at different temperatures and under a total pressure of 1 bar. To ensure maximum gas purity, especially concerning water and carbon oxides, the He carrier gas was passed through a liquid N_2_ trap before entering the cell. The spectra at both edges have been background-subtracted by fitting the pre-edge with a straight line. The spectra at the Sn M_4,5_-edges ere normalized at unit absorption at 508 eV. The spectra at the O K-edge are shown without further manipulation, except in cases where it is explicitly stated differently.

### Theoretical Calculations

DFT calculations were performed for water-loaded SnO_2_(110) interfaces with the Vienna ab initio simulation package (VASP 5.4.1)^37,38^ using the GGA approach. The PBE exchange-correlation was applied.^39,40^ To include van der Waals interactions, the dDsC dispersion correction method is employed (PBE-dDsC).^41−43^ The projector augmented wave approximation (PAW)^44^ is used to describe the interaction between the valence electrons and the atom cores. Sn and O were treated with 4 and 6 electrons in the valence, respectively. Bulk SnO_2_ was optimized using a plane wave kinetic energy cutoff of 1000 eV and a Γ-centered *k* point grid of at least 12 × 12 × 12. For SnO_2_, the optimized cell parameters are (*a* = *b*), 4.82 Å in the *a*- and *b*-direction and 3.24 Å in the *c*-direction, respectively. Subsequently, a 4-layered (4×4) slab is constructed with SnO_2_(110) termination at the top and bottom of the *z*-direction (Supporting Information, Figure S1). On such slabs, water chemisorption and the fate of methane are studied. All bulk SnO_2_(110) slabs were structurally relaxed using a plane wave cutoff energy of 450 eV, and Brillouin zone sampling was restricted to the Γ-point. A Gaussian smearing^45^ of 0.05 eV was applied to improve convergence. Additionally, the convergence criterion for the electronic self-consistent field (SCF) problem is set to 10^–5^ eV for cell optimizations, and structures are relaxed until all forces are below 0.03 eV/Å. A vacuum of at least 18 Å in the *z*-direction is present for all optimized models.

Ab initio thermodynamics is used to calculate adsorption/desorption equilibria (Figure 5c) or construct phase diagrams ranking different hydrated SnO_2_(110) surface slabs (see Figure S7). The pressure and temperature dependence of the equilibrium is introduced via chemical potential of the species in the gas phase. For example, for a compound C, which can adsorb and desorb from a SnO_2_ surface slab, this can be written as follows
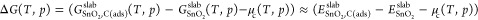
1

In these equations, we can approximate the free-energy difference with and without adsorbed species C with the zero-point corrected energy difference (*G* = *E* + *PV* – *TS* ≈ *E*). Then, we neglect the PV term as well as the TS term for the surface slabs. The chemical potential of species C can be written as the following sum
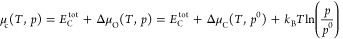
2Here, *E*_C_^tot^ represents the zero-point corrected energy, while Δμ_C_(*T*, *p*) can be calculated via the tabulated standard chemical potentials Δμ_C_(*T*, *p*^0^) at *p*^0^=1 bar.^46^ The standard chemical potentials Δμ(*T*, *p*^0^) at *p*^0^= 1 bar can be calculated from enthalpy and entropy; for example
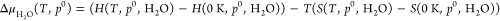
3Equilibria between adsorption and desorption can then be approximately calculated from eq 1 assuming Δ*G*(*T*, *p*) = 0 and visualized in a (*T*,*p*) diagram.

## Results and Discussion

### Sample Characterization

The SnO_2_ nanoparticles were prepared as described in the method section. The phase purity of the nanoparticles was checked before the XAS experiment by means of X-ray powder diffraction. The pattern (Figure S2, Supporting Information) shows the characteristic reflections of cassiterite SnO_2_ only. The crystallite size was estimated according to the Scherrer equation, showing an average grain dimension of 3.5 nm. The specific surface area, measured as described in the materials and methods’ section, is 117 m^2^ g^–1^; considering the average grain size and assuming particles with a spherical shape and cassiterite bulk density, the surface area value is indicative of a high degree of aggregation.

Notwithstanding the assumptions made in this estimate, the aggregation is independently confirmed by HRTEM results as shown in Figure S3, where a representative HRTEM image of the as-prepared SnO_2_ sample is shown. The oxide particles are indeed highly agglomerated, as individual objects cannot always be singled out. Almost all particles exhibit roundish contours, and the average particle size can be estimated (assuming an almost spherical form or, at least, a pebble-like shape) to be in the 3–5 nm range, in excellent agreement with the XRPD results, thus allowing the definition of nanoparticles. Moreover, the SnO_2_ nanoparticles are very thin (supporting then a pebble-like shape) and closely packed, giving rise to frequent fringe patterns, either “normal” and due to thickness or of a Moiré character. The analysis of both fringes and fast Fourier transform (FFT) patterns of the fringes themselves (Figure S3 inset) confirms the presence of the crystallographic planes belonging to the (110) of cassiterite with *d*_(*hkl*)_ = 0.33 nm (ICDD card no. 01-071-0652). The feature was revealed (i) onto many different portions of the sample and (ii) for many different parts of the sample.

The single phase of the nanoparticles is confirmed by the Raman spectrum reported in Figure S4. The spectrum is dominated by a sharp and intense component at ∼630 cm^–1^, in addition to two minor spectral components at ∼475 cm^–1^ (sharp, but with low intensity) and at ∼725 cm^–1^ (broad and low in intensity). All observed components can be ascribed, on the basis of both their spectral features and literature data,^47^ to SnO_2_ Raman modes. In particular, components at 630, 475, and 725 cm^–1^ can be attributed to the A_1g_, E_g_, and B_2g_ vibrational modes, respectively. As the Raman spectrum becomes totally opaque in the high-frequency region, no other spectral components could be singled out.

The FTIR spectrum of the as-synthesized SnO_2_ sample (Figure S5, Supporting Information) shows as the main feature a broad band in the 700–400 cm^–1^ region, which is attributed to Sn–O and Sn–O–Sn stretching vibrations.^48^ Moreover, a broad band in the 3700–3000 cm^–1^ range is attributed to stretching vibrations of hydroxyl groups and physisorbed water, mutually interacting by hydrogen bonding. The corresponding bending counterpart can be appreciated at 1640 cm^–1^. It should be noted that FTIR spectra were recorded on the as-synthesized powder, and hence, the presence of abundant surface hydration might cover any signals of free OH groups. No peaks characteristic of organic contaminants (e.g., CH*_x_* or C=O stretching vibrations) can be observed. Thermogravimetric analysis (TGA, Figure S6, Supporting Information) shows a loss of physisorbed water at *T* < 100 °C and a broader feature starting at about 400 °C accounting for an overall weight loss of about 4%; the latter can be related, in agreement with FTIR results and previous literature, mainly to the loss of chemisorbed water.^49^ As a result, an average surface density of ca. 5 OH groups per nm^2^ can be estimated,^50^ which is in good agreement with previous studies.^49^ It should be noted that TGA shows that in the temperature range between 100 and 360 °C, the weight loss is the smallest, amounting to ca. 0.1%. To better understand this fact, DFT calculations of adsorption energy were performed. We found that on the bare SnO_2_(110), water can be adsorbed dissociatively (Figure S7, Supporting Information), with an average chemisorption energy of −161.6 kJ/mol (PBE-dDsC) per water molecule (at PBE, −140.1 kJ/mol). The first H_2_O molecule chemisorbs strongly onto our bare SnO_2_(110) model system, with a reaction energy of −164.9 kJ/mol (θ_H_2_O_:0 → 1/8, where θ_H_2_O_ is the coverage degree). The chemisorption energy of the last molecule is −159.9 kJ/mol (θ_H_2_O_:7/8 → 8/8). There is thus about 5 kJ/mol difference between the first and the last H_2_O during chemisorption, and the average chemisorption energy of 8 water molecules lies in between (−161.7 kJ/mol).

More loosely bound water (physisorbed water) can further adsorb on the SnO_2_(110) already covered with chemisorbed water, with adsorption energies of −78.0 kJ/mol (PBE-dDsC, at PBE; −60.8 kJ/mol) for the first layer and −49.6 kJ/mol (PBE-dDsC, at PBE; −33.6 kJ/mol) for the second layer (Figure S7), characteristic of hydrogen-bonded water. These different adsorption energies are closely reflected in the TGA curve: The weight loss for *T* < 100 °C is associated to the first and second layer of physisorbed water (and additional layers); above 100 °C, the weight loss is associated with the desorption of chemisorbed water, which happens at a much slower rate in the TGA in the 100 < *T* < 360 °C range.

### Operando Soft-XAS Experiment

The XAS experiment was performed at the O K- and Sn M_4,5_-edges using the experimental ambient-pressure XAS cell shown in Figure 1.^36,51^ In order to clean the sample surface from the physisorbed water, after being mounted in the reaction gas XAS cell, the sample was heated up to 300 °C in flowing He at 20 sccm and then cooled down to room temperature.

Figure S8 (Supporting Information) compares the spectra obtained for the sample as it is and during the initial thermal treatment; that obtained in vacuum conditions is also shown for better reference.

The peak at ca. 531 eV is due to molecular oxygen (see Figure S9, Supporting Information), and it is reasonably due to desorption from the gas pipeline. The dip at ca. 531 eV is due to small oxygen impurities in the beamline, and it is better discussed in the Supporting Information (Figure S8); here, we note that it can be safely ignored. Between 533 and 543 eV, the spectra at *T* > 100 °C show a series of features. In particular, the peaks at ca. 534, 536.1, and 537.2 eV are due to the appearance of molecular water in the gas phase, confirming that in the temperature range 100 < *T* < 300 °C, the desorption of physisorbed water takes place. Comparing the spectrum obtained at room temperature, in flowing He and after this thermal treatment, with that obtained in rough vacuum (10^–3^ torr) before the thermal treatment demonstrates that SnO_2_ is now free of physisorbed water, in agreement with the TGA measurements. However, TG and DFT calculation show that dissociatively chemisorbed water is still present at the surface, and therefore, the SnO_2_ nanoparticles are terminated by hydroxyl groups. For the sake of conciseness, in the following, this will be referred to as the “clean SnO_2_ surface”. In addition, the spectrum in rough vacuum shows again a small peak between 530 and 531.5 eV due to desorption of O_2_ from the cell pipeline, demonstrating the sensitivity of the O K-edge spectra to small amounts of oxygen-containing species in the gas phase. It is also noted that comparison with literature data shows a small broadening of the spectral features at the O K-edge of the SnO_2_ sample when compared to literature data.^52^ This may be caused by the large amount of uncoordinated atoms that are present at the surface due to the nanosize of our sample.

**Figure 2:**
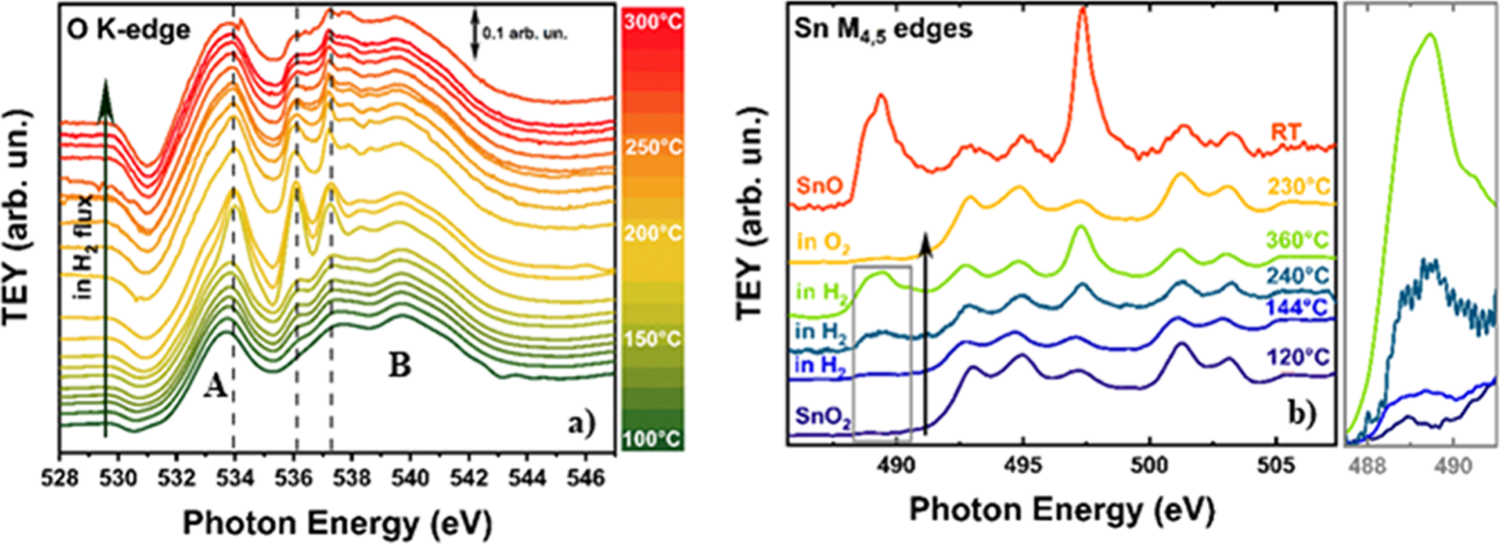
(a) Evolution of the O K-edge spectra of the SnO_2_ nanoparticles in flowing H_2_ as a function of temperature. The spectra are shifted along the *y* axis for the sake of better clarity. The vertical dotted lines mark the position of the main peaks of water in the gas phase. Peaks A and B are the main peaks of SnO_2_, whose origin is described in the text. (b) Sn M_4,5_-edges of the SnO_2_ nanoparticles at different temperatures and in different conditions. The spectrum of standard SnO is shown for reference, and the spectra are shifted along the *y* axis as in a. The spectrum after the reoxidation in O_2_ at 230 °C is also shown as a yellow line. The region between 487.5 and 492 eV for the SnO_2_ nanoparticles at 120, 144, 240, and 360 °C in H_2_ is evidenced for better reference.

The sample was then heated while flowing a mixture of He (20 sccm) and H_2_ (5 sccm). The results are shown in Figure 2a (O K-edge) and b (Sn M_4,5_-edges).

Due to the 1s character of the initial state, at the O K-edge, the Δ*l* = ±1 dipole selection rule allows transition to empty states with the p character only. In addition, the localized nature of the core hole in the 1s orbital that is formed after photon absorption projects the density of the final states onto states with the O character only.^53^ The two intense peaks in the O K edge spectrum are then due to transitions to O 2p states hybridized with Sn 5s states at the bottom of the conduction band (peak A in Figure 2a) and to transition to antibonding O 2p states hybridized with Sn 5s and 5p states in the conduction band (peak B in Figure 2a), respectively.^52^ According to the same type of considerations, the final states at the Sn M_4,5_-edges are of Sn p and f character, with the spin–orbit coupling splitting the initial states in 3d_3/2_ (M_4_) and 3d_3/2_ (M_5_), respectively. The Sn M_4,5_-edge spectrum then maps the PDOS of the empty Sn 5p and 5f character in the conduction band.^53^

At 100 °C, the O K-edge spectrum shows a close resemblance with that of a clean SnO_2_ nanoparticle surface. Above this temperature, some additional peaks at ca. 534, 536.1, and 537.2 eV start to appear, with increasing intensity with increasing *T*. These peaks are strictly corresponding to that of H_2_O in the gas phase (Figure S9). In this case, we start with a clean surface, without physisorbed water, and in this temperature range, where we demonstrated that desorption of chemisorbed water cannot occur, these additional peaks can only be attributed to a reduction by H_2_ of the SnO_2_ surface to produce water in the gas phase. To further investigate this point, a spectrum at the Sn M_4,5_-edges was acquired in flowing H_2_ at ca. 144 °C. A close inspection of this spectrum in the region between 487.5 and 492 eV (inset in Figure 2b) shows the presence of an additional peak, which, by comparison with the spectrum of reference SnO, can only be attributed to the formation of Sn(II), thus confirming the reduction (see Table S1 in the Supporting Information).

At the O K-edge, the additional peaks at ca. 534, 536.1, and 537.2 eV show a sudden and dramatic boost at ca. 190 °C, showing that the rate of the reaction producing water becomes conspicuous. Then, a striking decrease of water production is found for *T* > 240 °C. Simultaneously, the Sn M_4,5_-edge data in Figure 2b show a noticeable increase of the extra peak at ca. 489 eV and an increase in intensity of the peak at 497.5 eV, both due to the formation of an increased amount of Sn(II). At ca. 240 °C, the spectrum at the Sn M_4,5_-edges shows that the reduction of SnO_2_ goes well beyond the surface, and a further increase of the reduction is found at 360 °C. A quantification of the thickness of the SnO layer can be obtained by measuring the area of the peak at 489 eV, which is only due to Sn(II), normalized by the area of the peaks at 492.5 and 494.5, which are almost only due to Sn(IV) (see Figure S10, Supporting Information). Taking into account that the mean probing depth λ of electrons in the total electron yield at 500 eV amounts to ca. 3.5 nm,^54^ the thickness of the reduced layer at 360 °C is ca. 1.5(5) nm, which corresponds to a width of at least 3–4 unit cells. This surface layer prevents further reduction of the SnO_2_ nanoparticles and avoids further production of water. Notably, as it is shown in Figure 2b, the reduction can be fully reversed by flowing O_2_ in the ambient-pressure XAS cell at *T* > 200 °C. The Sn M_4,5_-edge spectrum obtained after this treatment shows that the spectral shape of SnO_2_ is fully recovered. To further confirm that H_2_O is present in the gas phase, we fitted the spectrum at 200 °C at the O K edge with a linear combination of the spectra of water in the gas phase and of the SnO_2_ nanoparticles in vacuum. The agreement, as shown in Figure S11 of the Supporting Information, is rather satisfactory.

After having demonstrated that in situ soft-XAS is successful (in real working conditions) in both detecting gas products of surface reaction(s) and the variations in the Sn oxidation state at the surface produced by these reaction(s), we consider the effects of hydrocarbons like CH_4_ and CH_2_CHCH_3_.

**Figure 3:**
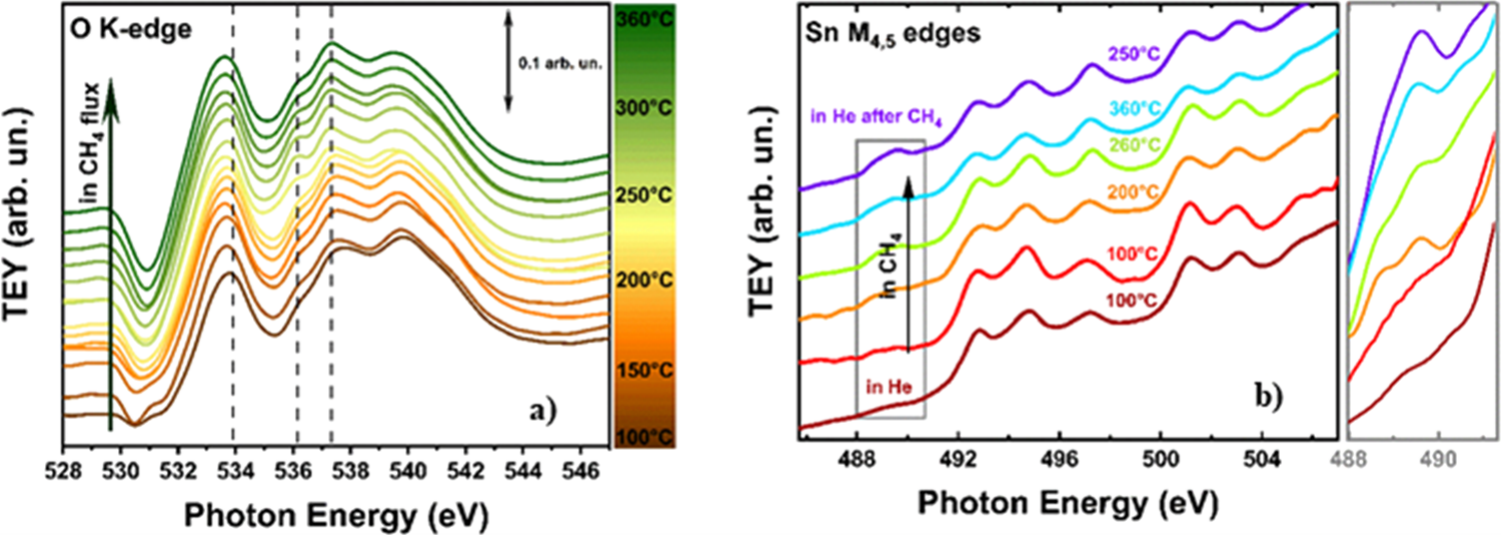
(a) Evolution of the O K-edge spectra of the SnO_2_ nanoparticles in flowing CH_4_ as a function of temperature. As in Figure 1, the spectra are shifted along the *y* axis for the sake of better clarity, and the vertical dotted lines mark the position of the main peaks of water in the gas phase. (b) Sn M_4,5_-edges of the SnO_2_ nanoparticles at different temperatures in flowing CH_4_. The spectra are shifted along the *y* axis as in a. The increase of the Sn(II) signature at 489 eV is shown on an enlarged scale for better reference.

Experiments with methane (He (20 sccm) + CH_4_ (5 sccm)) were conducted in the 100 ≤ *T* ≤ 360 °C temperature range. Before running the experiments, the sample was fully oxidized as described above. The results at the O K-edge are shown in Figure 3a. At 100 °C, the O K edge spectrum corresponds to a clean SnO_2_ surface, free of physisorbed water. This is again in agreement with the TGA measurements of Figure S6, showing that the rate of water desorption is minimum in the 100 ≤ *T* ≤ 360 °C temperature range. At *T* = 120 °C, the CH_4_ flux produces an appearance of the signatures of water in the gas phase.

These signatures show an increase at *T* ≥ 240 °C, with a maximum at 310 °C, and then a decrease when going from 310 to 360 °C. The spectra at the Sn M_4,5_-edges are shown in Figure 3b. Here, we note that signatures of Sn(II) start to appear after the treatment in CH_4_ at *T* = 120 °C, with an intensity that is increasing by increasing *T*, as it is shown by the inset in Figure 3b.

**Figure 4:**
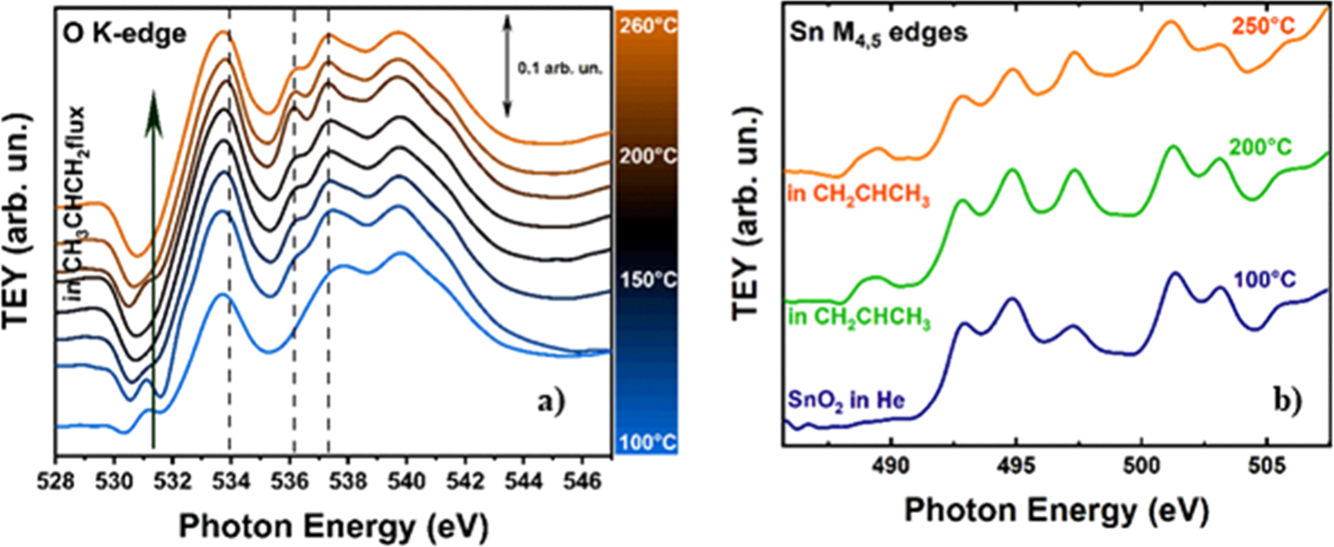
(a) Evolution of the O K-edge spectra of the SnO_2_ nanoparticles in flowing CH_2_CHCH_3_ as a function of temperature. As in Figure 1, the spectra are shifted along the *y* axis for the sake of better clarity, and the vertical dotted lines mark the position of the main peaks of water in the gas phase. (b) Sn M_4,5_-edges of the SnO_2_ nanoparticles at different temperatures in flowing CH_2_CHCH_3_. The spectra are shifted along the *y* axis as in a.

Results with CH_2_CHCH_3_ (He (20 sccm) + CH_2_CHCH_3_ (5 sccm)) are analogous. Also in this case, before running the experiment, the sample was fully oxidized by flowing O_2_ at 200 °C. The measurements were performed in the 100 ≤ *T* ≤ 260 °C temperature range. The pertinent spectra at the O K-edge are shown in Figure 4a. Again, we observe that the initial conditions correspond to that of an almost clean SnO_2_ and that the signatures of water in the gas phase appear for *T* ≥ 120 °C, reaching a maximum at 200 °C and then decreasing at higher temperatures. The spectral intensity of the water peaks is larger for propylene than for methane, in agreement with the higher reactivity of propylene. Again, at the Sn M_4,5_-edges (Figure 4b), the hydrocarbon flow induces an appearance of the peaks of Sn(II).

An important point to note is that with both methane and propylene, signatures of carbon oxides are never detected by soft-XAS, even if the spectra of both CO and CO_2_ show very large pre-edge peaks at the O K-edge^55^ that are likely to show up in the spectra (see Figure S9), even if these compounds are present in very low concentrations. It is also noted that the cross section of CO and CO_2_ at the O K-edge is at least five times larger than that of water,^56,57^ indicating that the possible formation of carbon oxides is well within the sensitivity of our soft-XAS experiment. The non-appearance of carbon oxide in the O K-edge spectra has the obvious meaning that hydrocarbons react with the clean SnO_2_ surface by directly providing electrons rather than by removing surface-oxygenated species. Likely, SnO_2_ behaves in these cases like an electron sponge, being able to easily accept and return electrons depending on the gas species that are close to the surface. This is also confirmed by the ease with which the reduced surface can be restored in its fully oxidized form. The electrons provided by reducing gases localize on Sn to form Sn(II) and eventually an SnO layer at the surface. Quantification of the thickness of the SnO layer using the same method described above for the reduction with H_2_ gives a figure of 0.6(2) nm at 360 °C in CH_4_ and 0.5(2) nm at 250 °C in CH_2_CHCH_3_ (see Figure S10, Supporting Information), which correspond to a width of 1–2 SnO_2_ unit cells in both cases. The thicknesses in CH_4_ and CH_2_CHCH_3_ are comparable despite the fact that the reduction is faster in CH_2_CHCH_3_ because the SnO_2_ nanoparticles have been exposed to methane at high *T* for a longer time.

### DFT Calculations

**Figure 5:**
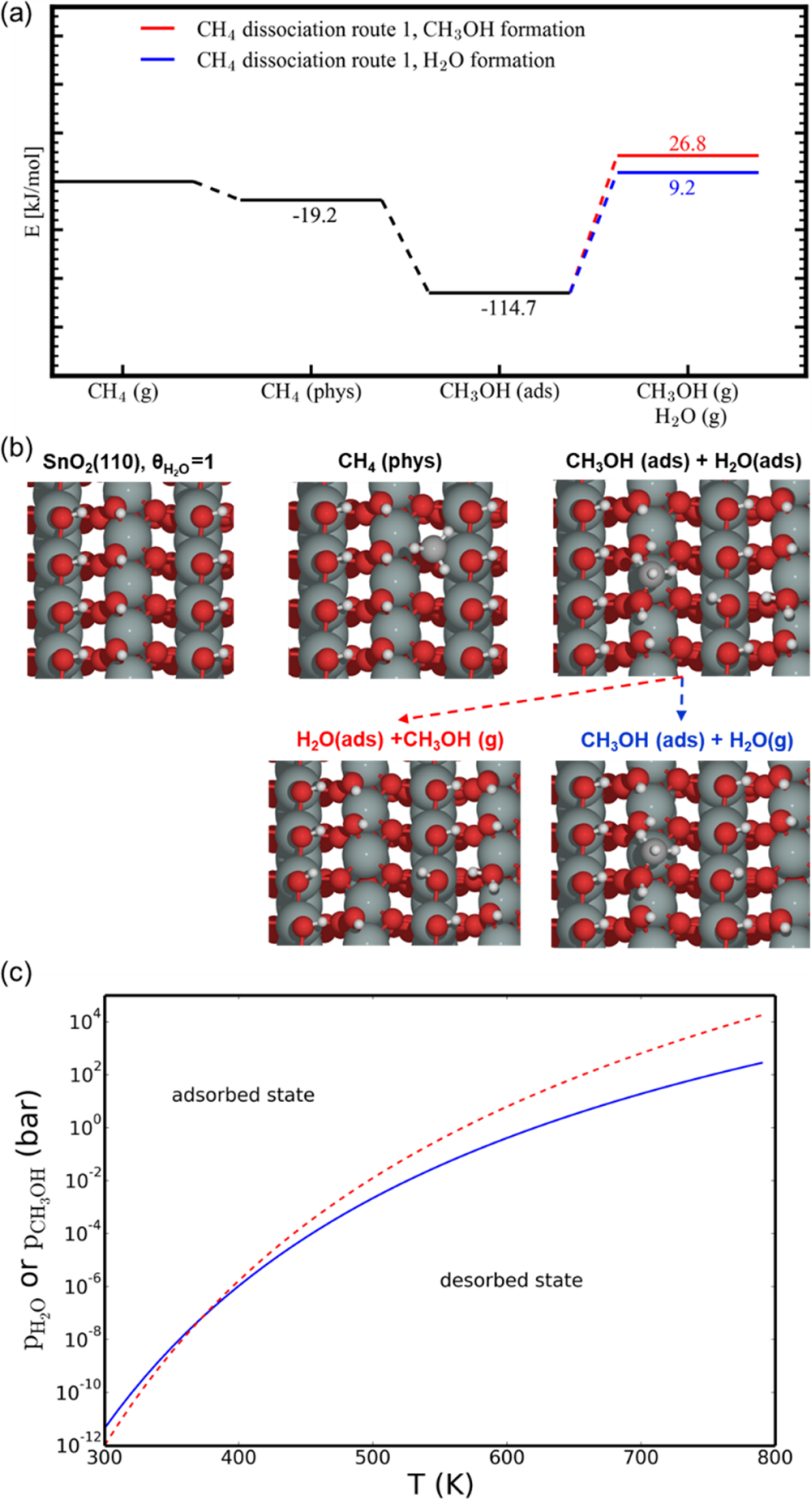
(a) Energy diagram for the production of CH_3_OH and H_2_O from CH_4_ on a fully hydroxylated SnO_2_(110) surface via CH_3_ deposition on a dangling Sn–OH bond forming adsorbed (ads) CH_3_OH and (b) schematic representation of the different states of SnO_2_(110) in the above energy diagram (a). Color code: Sn (green), O (red), C (gray), and H (white). (c) Calculated adsorption/desorption equilibrium curves for H_2_O (blue) and CH_3_OH (red).

**Figure 6:**
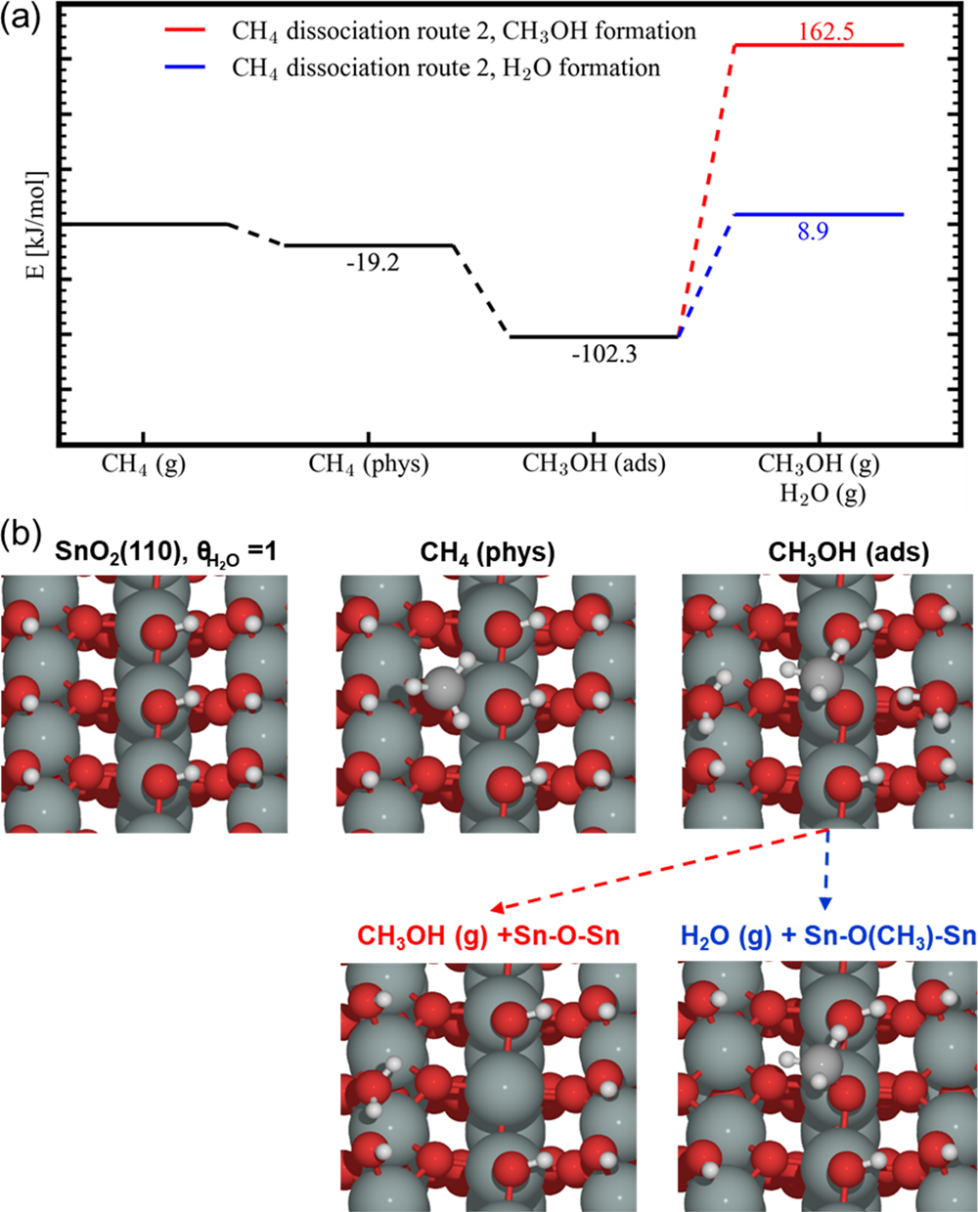
(a) Energy diagram for the production of CH_3_OH and H_2_O from CH_4_ on a fully hydroxylated SnO_2_(110) surface via CH_3_ deposition on a Sn–O–Sn bridge forming adsorbed (ads) CH_3_OH and (b) schematic representation of the different states of SnO_2_(110) in the above energy diagram (a). Color code: Sn (green), O (red), C (gray), and H (white).

Experimental evidence described above allowed us to propose different dissociative hydrocarbon routes that have been tested by DFT calculations. For the sake of simplicity but without any loss of generality, we studied the dissociative adsorption of CH_4_ on fully hydroxylated SnO_2_(110) surfaces (θ_H_2_O_ = 1), mimicking the predominantly exposed clean surface in the experiments (Figures 5 and 6). These (110) surfaces consist of Sn^4+^ and have both dangling Sn–OH and bridged Sn–OH–Sn groups (Figure 5). Furthermore, they are fully H-saturated, and therefore, the reaction with CH_4_ takes place via physisorption of CH_4_ (−19.2 kJ/mol) followed by C–H cleavage forming adsorbed CH_3_OH. Here, two different reaction possibilities are tested, forming the adsorbed CH_3_OH either via CH_3_ addition to a dangling Sn–OH bond or to a bridged Sn–OH–Sn bond, represented in Figures 5 and 6, respectively. The most stable reorganized structures after O–CH_3_ bond formation are at −114.7 and −102.3 kJ/mol (see Figures 5 and 6). From these states, the desorption of CH_3_OH and H_2_O was considered. This means that when the adsorption energy is similar, CH_3_OH desorption from the SnO_2_(110) surface takes place at higher temperatures compared to H_2_O. In the case of water, we find desorption energies of 124 and 111 kJ/mol (see Figures 5 and 6). Within experiment, it is clear that H_2_O formation happens from 100 °C after exposure of the SnO_2_ nanoparticles to CH_4_ (see Figure 2), and this agrees with the computational result that reaction with CH_4_ lowers the H_2_O adsorption energies (Figures 5 and 6). In particular the adsorption energy is lowered from strongly chemisorbed H_2_O (−161.6 kJ/mol) to physisorbed H_2_O (−124 and −111 kJ/mol), making it easier for H_2_O to desorb.

To estimate the feasibility of C–H activation on fully hydrated SnO_2_, we can use the Evans–Polanyi principle, stating that there is a relationship between the activation energy and the energy of formation, and comparisons can be made with similar reactions. Such relationships for CH_4_ activation are universal for different materials, e.g., oxides, zeolites, MOFs, etc. and have been determined computationally via screening of various materials.^58^ In particular, if the transition state (TS) has a radical character, the activation barrier relates to the hydrogen adsorption energy via the relationship *E*_TS_ = 0.75*E*_H_ + 1.09 [eV], and *E*_H_ is calculated based on the energy difference of the active site before and after hydrogenation in the case of an oxide
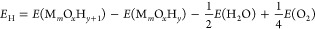
4

Using this formula and scaling relations, we can estimate the reaction barrier for CH_4_ activation via a radical-like intermediate. For the fully hydrated SnO_2_(110) model with chemisorbed H_2_O (θ_H_2_O_ = 1), we identified reaction barriers of about 110.7 ± 1.1 kJ/mol. These are realistic reaction barriers resulting in feasible turnover rates at higher temperatures (>150 °C). Methanol probably reacts further on the surface as it is not observed in the products.

**Figure 7:**
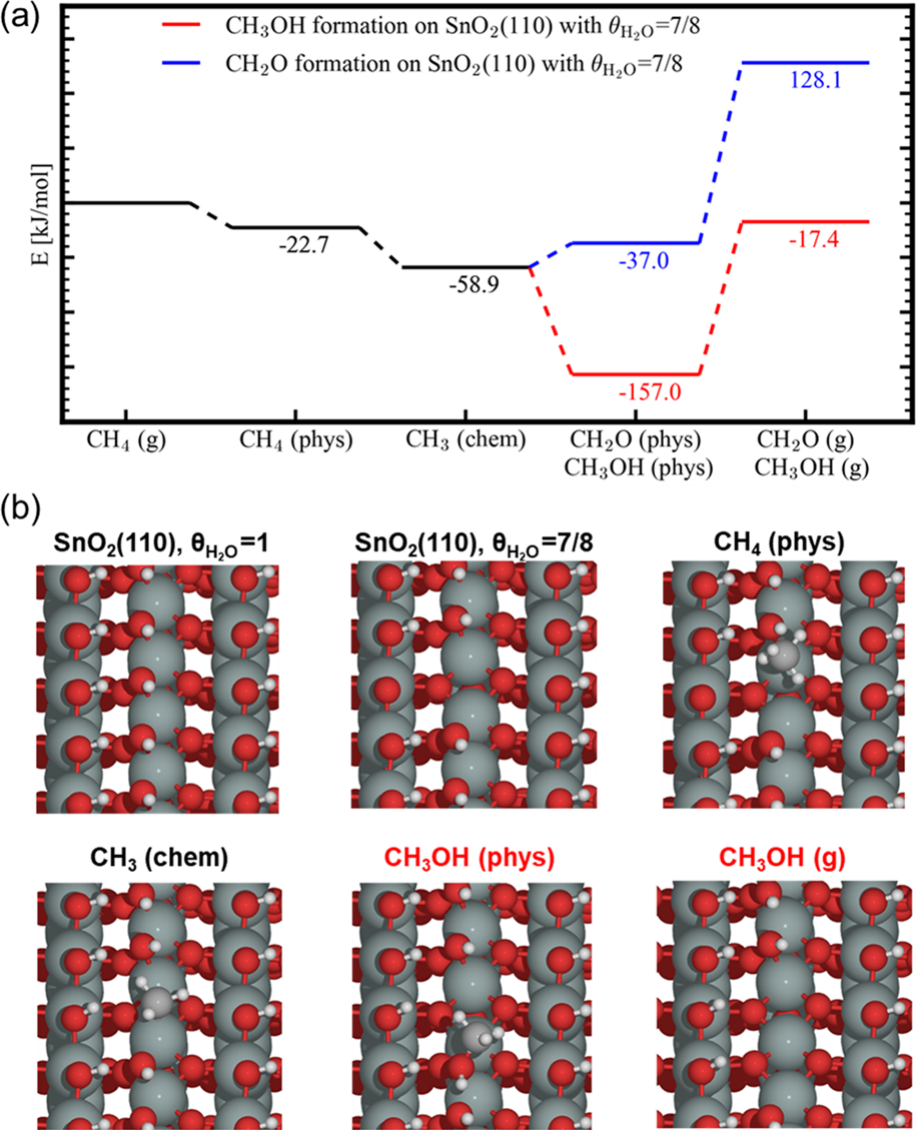
(a) Energy diagram for the production of CH_3_OH or CH_2_O from CH_4_ on a hydroxylated SnO_2_(110) surface (H_2_O coverage of 7/8) via CH_3_ chemisorption (chem) on the bare Sn site followed by the formation of physisorbed (phys) CH_3_OH or CH_2_O and (b) schematic representation of the different states of SnO_2_(110) in the above energy diagram (a). In this case, the formation of H_2_O from the physisorbed CH_3_OH state is more endothermic than the formation of gas-phase CH_3_OH (energy level, −13.1 kJ/mol). Color code: Sn (green), O (red), C (gray), and H (white).

In another scenario we investigated, the SnO_2_(110) surface is not fully hydroxylated, and therefore, one water molecule was removed (θ_H_2_O_ = 7/8), creating a free Sn site and an O site without hydrogen (Figure 7). In this case, methane is slightly exothermically physisorbed at the free Sn site. The nearby oxygen removes a hydrogen, and the remaining −CH_3_ group becomes chemisorbed, forming a Sn–CH_3_ bond. This step is exothermic with about 59 kJ/mol compared with methane in the gas phase. In the next step, a methoxy group is formed, and water leaves the surface, reforming a free Sn site.

This reaction is exothermic with −157 kJ/mol and shows that the CH_3_OH group is strongly physisorbed onto the surface. Since the active site is reformed, the reaction is autocatalytic and will stop once all available sites are sterically blocked by methanol or methoxy groups.

These results clearly show that both water and methanol cannot be formed unless the desorption energies are lowered further, for example, via extra surface reactions with CH_4_. Remark that as soon as bond-deficient Sn sites at the surface are formed, species like Sn–CH_3_ can appear at the surface. Through such an electron-rich covalent bond, surface Sn(IV) gets eventually reduced. The fact that a Sn(II) signature is observed and that only 1–2 layers of SnO were found experimentally further support this latter reaction sequence.

The results described above can be further discussed in the framework of the gas sensing mechanisms by SnO_2_ proposed in the literature. The sensor response is explained by changes of the electric surface potential resulting from “ionosorption” of gaseous molecules (ionosorption model) or by changes in the oxygen stoichiometry, that is, by the variation of the number of (sub) surface oxygen vacancies and their ionization (reduction–reoxidation mechanism).^13^ A very recent work concerning the mechanisms of gas sensing toward ethanol by SnO_2_ concluded that the response is correlated with the number of oxygen vacancies, the nature of the adsorbates, and the presence of surface hydroxyl groups, thus supporting the reduction–reoxidation mechanism.^59^ The present work makes a further step forward in this direction: Reducing gases such as hydrocarbons can directly provide electrons to SnO_2_ nanoparticles via cleavage of the C–H bond(s). These electrons are at least partially localized on Sn to produce Sn(II); the reduced layer eventually forms a monolayer (or a couple of monolayers) of SnO at the surface. In principle, in this mechanism, the presence of oxygen vacant sites at the surface is not required. On the contrary, our results show that the presence of hydroxyl species is decisive for the dissociative chemisorption of hydrocarbons.

## Conclusions

In conclusion, it is demonstrated that soft-XAS can be used to probe, in the same experiment, the products of a surface heterogeneous reaction both in the gas phase and, simultaneously, on the surface. In particular, we experimentally demonstrated that reducing gases directly react with the hydroxylated SnO_2_ surface, removing water and reducing Sn(IV) to Sn(II). The reduction is larger for H_2_ than for hydrocarbons and is larger for unsaturated hydrocarbons than for alkanes. Hydrocarbons act as a reducing agent without the production of carbon oxides. This fact indicates that methoxy- or methyl-like species at the surface can be formed, as indicated by detailed theoretical DFT investigation on the reaction mechanism for the reduction with CH_4_. As schematically represented in Figures 5–7, the surface reaction involves cleavage of the C–H bond of the incoming gas and subsequent reaction with OH groups on the surface to form H_2_O and methoxy- or methyl-tin species on the surface. Because of this reduction, more loosely bound or physisorbed H_2_O is formed, eventually leaving the SnO_2_ surface. All data presented in this work reveal that the hydroxyl groups that are present at the SnO_2_ surface play a crucial role in the adsorption properties: This evidence can be the basis for a better understanding of both sensing and catalytic properties of SnO_2_ and, more generally, of oxide-based materials.

A final comment is due concerning the transferability of the present results to other systems. With a typical setup used at a synchrotron radiation beamline for the O K-edge, all edges within the 300 and 1200 eV energy range can be reached. This means that (i) all L_2,3_ edges of the 3d transition metals, (ii) all M_2,3_ edges of the 4d transition metals, (iii) all N_2,3_ edges of the 5d transition metals, and (iv) the M_4,5_-edges of the lanthanides from Ce to Tb can be probed in the same experiment. Although these are just few illustrations among the variety of edges that can be probed simultaneously with the O K-edge, these examples show that the methodology here described can in principle be applied to the vast majority of cases that are of interest when a solid is put in contact with a gas phase.

## Supporting Information Available

(Figures S1–S11) Chosen SnO_2_(110) model system; XRD pattern; HRTEM; Raman and FTIR spectra of the SnO_2_ nanoparticles; TGA curve; water loadings for SnO_2_ in the DFT model; XAS spectra under the heating process; XAS spectra of the gaseous species relevant to the work; quantification of the reduced SnO_2_ layer under the reducing gases; fitting of the O K-edge spectrum at 200 °C under H_2_; (Table S1) peak assignment at the Sn M_4,5_-edges (PDF)
