# Supplementary material for: Understanding Solid–Gas Reaction Mechanisms by Operando Soft X-Ray Absorption Spectroscopy at Ambient Pressure
Source: J Phys Chem C Nanomater Interfaces. 2020 Jun 5;124(26):14202–12. doi: 10.1021/acs.jpcc.0c02546 (PMC8008446; doi:10.1021/acs.jpcc.0c02546)
Supplement: Supplementary file 1 — jp0c02546_si_001.pdf [file jp0c02546_si_001.pdf]

# Understanding Solid-Gas Reaction Mechanisms by Operando Soft X-Ray Absorption Spectroscopy at Ambient Pressure

*Luca Braglia[1], Martina Fracchia[2§], Paolo Ghigna[2,4*], Alessandro Minguzzi[3,4*], Daniela Meroni[3,4], Raju Edla[1], Matthias Vandichel[5*], Elisabet Ahlberg[6], Giuseppina Cerrato[7], Piero Torelli[1]*

[1]CNR- Istituto Officina dei Materiali, TASC, Trieste, Italia
[2]Dipartimento di Chimica, Università di Pavia, V.le Taramelli 13, I-27100, Pavia, Italy
[3]Dipartimento di Chimica, Università degli Studi di Milano, Via Golgi 19, 20133 Milan, Italy
[4]INSTM, Consorzio Interuniversitario per la Scienza e Tecnologia dei Materiali, Via Giusti 9, 50121 Firenze, Italy
[5]Department of Chemical Sciences and Bernal Institute, Limerick University, Limerick, Ireland
[6]Department of Chemistry and Molecular Biology, University of Gothenburg, Kemigården 4, SE-412 96 Gothenburg, Sweden.
[7]Department of Chemistry and NIST Interdipartimental center, Università degli Studi di Torino, via P. Giuria, 7, 10125 Torino (Italy)

## SUPPORTING INFORMATION

---

§ Luca Braglia and Martina Fracchia share the role of first Author

* Corresponding Authors

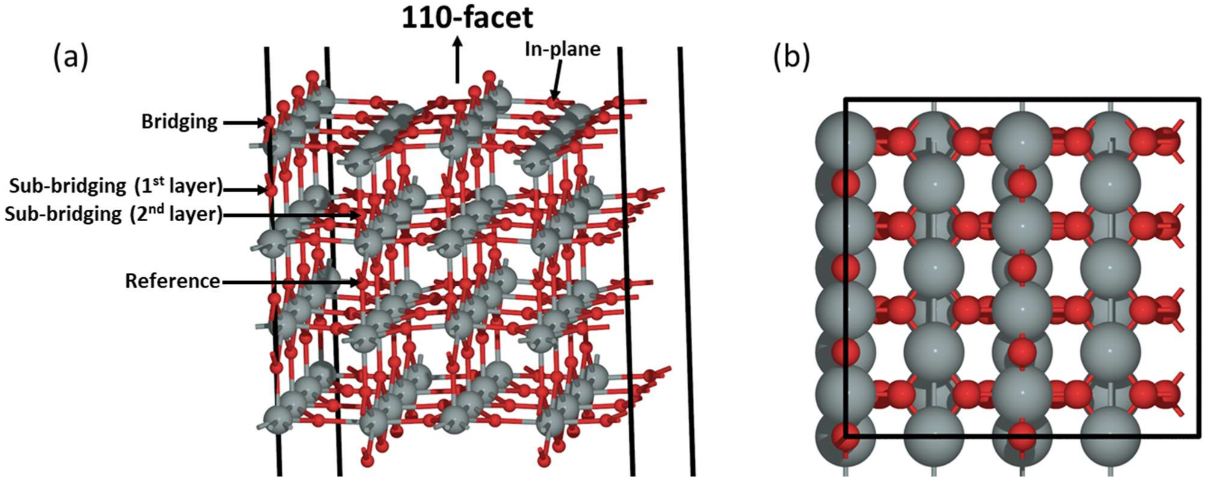


Fig. S1. Chosen $SnO_2(110)$ model system; (a) side view illustrating the position of the reference oxygens and assignment of other oxygen atoms; (b) top view.

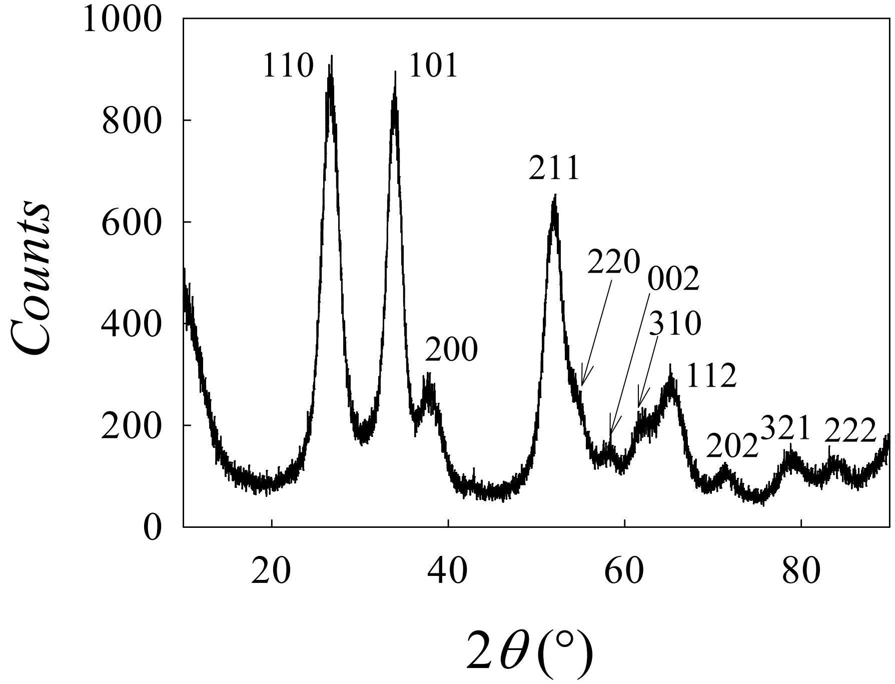


Figure S2. Powder diffraction pattern of the $SnO_2$ nanoparticles, indexed according to the cassiterite crystal structure.

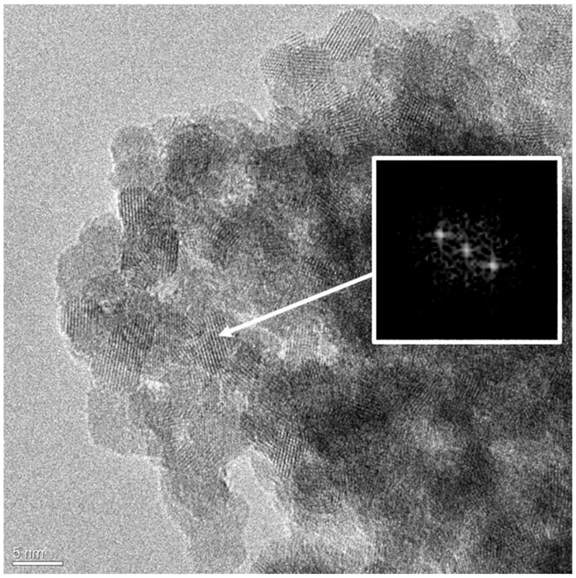


Figure S3. HRTEM image and FFT pattern (inset) of a portion of $SnO_2$ samples (scale bar: 5 nm).

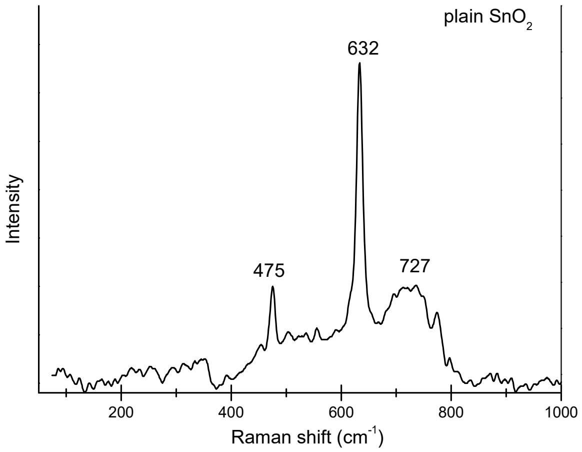


Figure S4. Raman spectra of the as prepared $SnO_2$ sample.

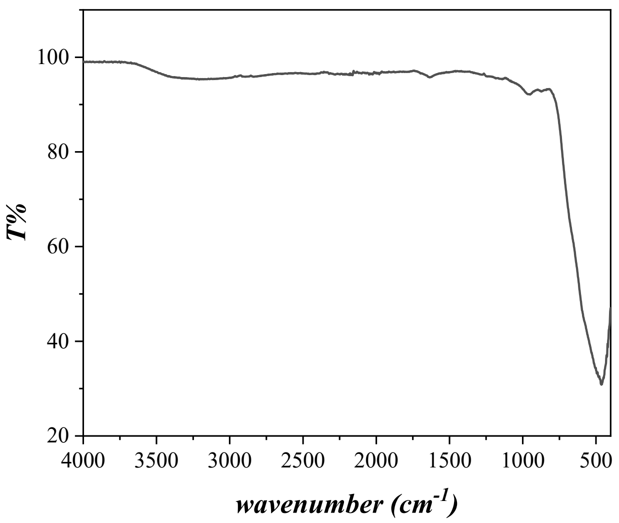


Figure S5. FTIR spectrum of the $SnO_2$ nanoparticles.

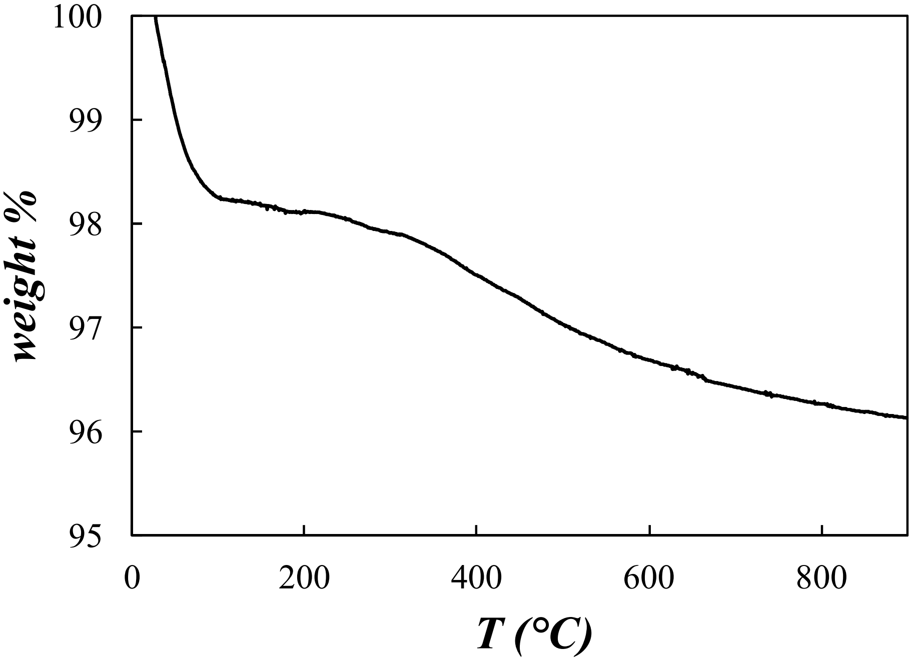


Figure S6. Thermogravimetric curve of the $SnO_2$ nanoparticle sample. The first weight loss between RT and 100 °C *ca.* is due to the desorption of physisorbed water, the second weight loss starting above 360 °C is mainly due to the loss of chemisorbed water.

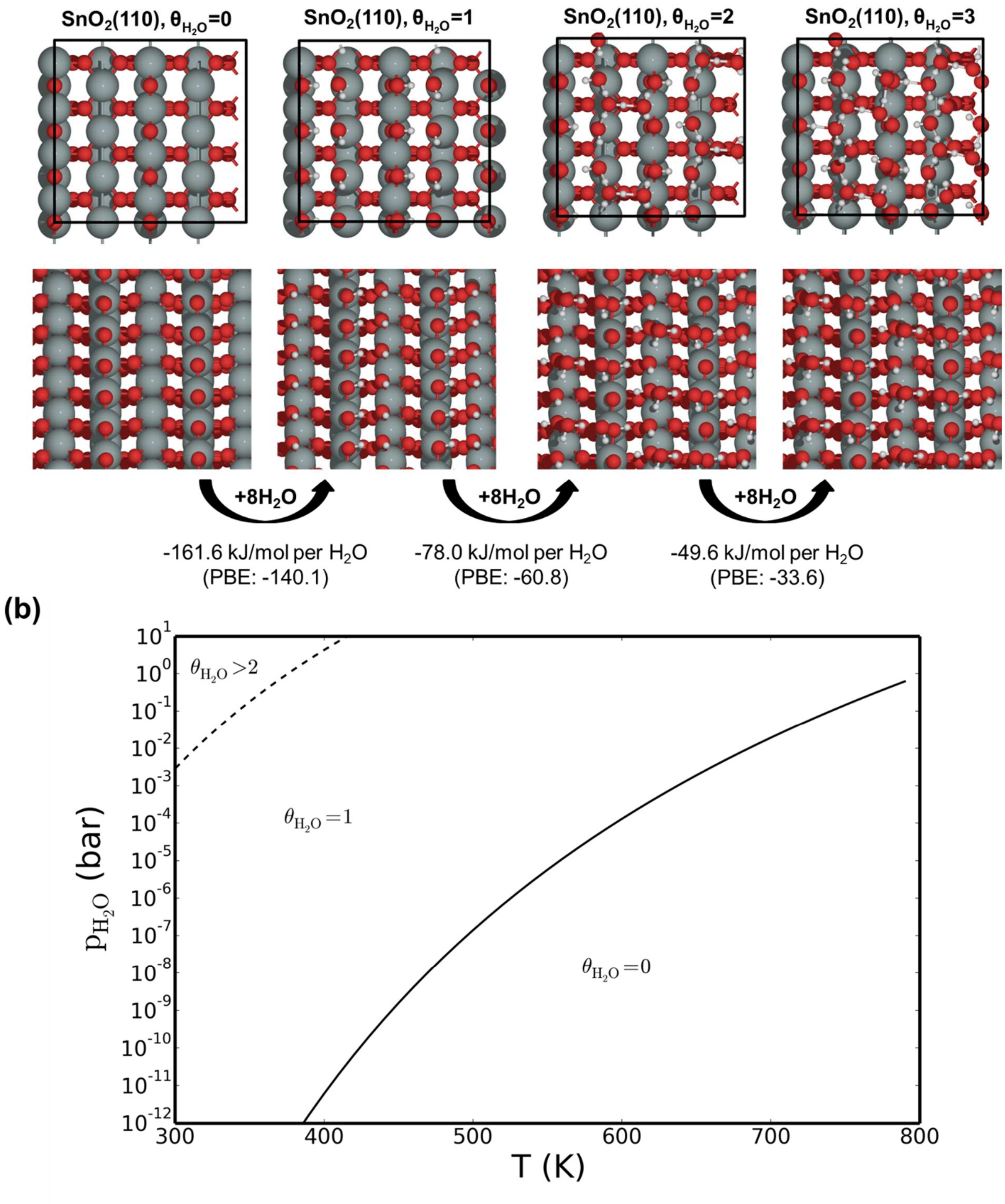


Figure S7. (a) Different water loadings of $SnO_2(110)$ with their differential water adsorption energy for each layer at PBE-dDSC in kJ/mol. Color code; Sn (green), O (red), C (gray), H (white); (b) phase diagram of the different model systems in function of $H_2O$ partial pressure and temperature.

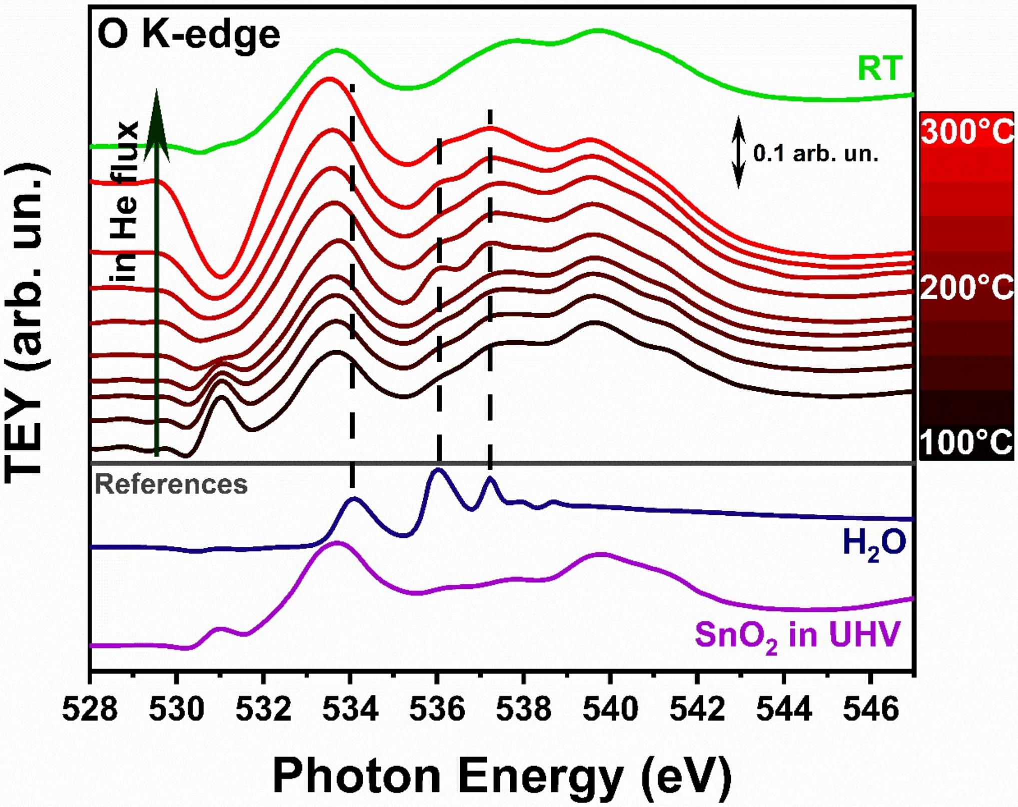


Fig. S8: O K-edge spectra of the $SnO_2$ nanoparticles in flowing He and at different temperatures between 100 and 300 °C. The green spectrum was obtained at RT after cooling down the sample while flowing He at 20 SCCM. The spectra of gaseous $H_2O$ and that of the nanoparticles in rough vacuum before the thermal treatment are shown for better reference. These last two spectra are normalised at unit absorption at 547 eV. Due to oxygen contamination of beamline optics (and also of the SiN membrane) the photon flux in the O K-edge energy region (like in the C and N region) is not flat and structureless but presents some oscillations due to the absorption of the contaminants. To correct the lineshape of the measured O K-edge different normalization procedure are possible[1], however it is not always possible to subtract completely from the spectra the contribution of the contamination, and the dip before the white line is a typical residual of the contaminant contribution to the spectrum as observed in a number of publications. This said, for our result it is not fundamental since we are looking at the modification of the spectrum with reaction (not at the exact lineshape) as it happens in a great number of publications on C or O K-edge.

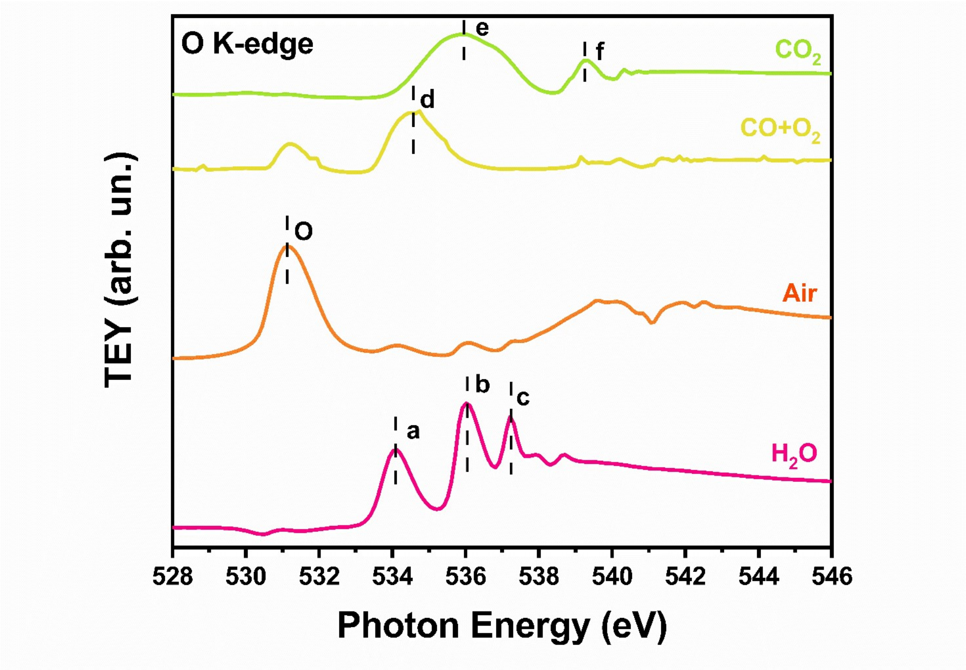


Fig. S9. O K-edge spectra of reference gases. The spectra are normalised at unit absorption at 548 eV, and shifted along the *y* axis for the sake of better clarity.

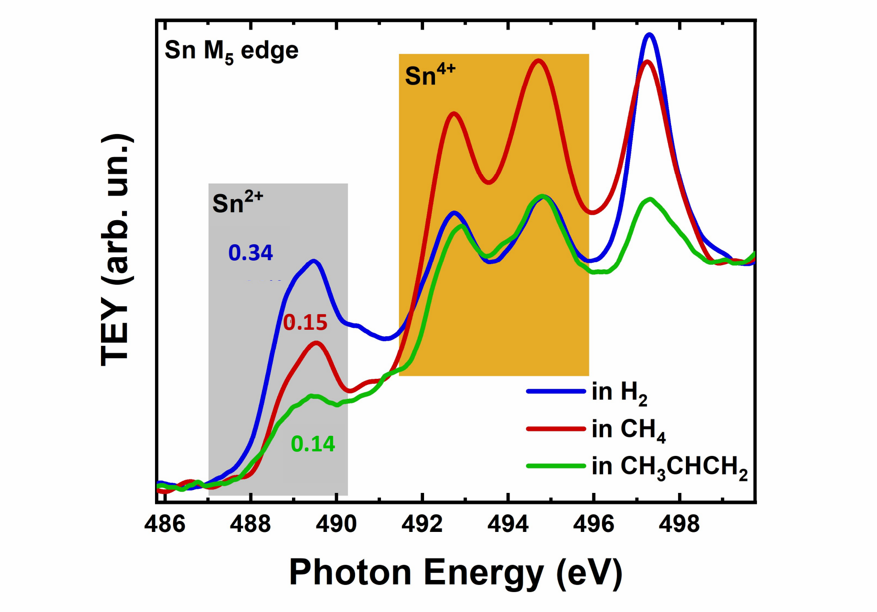


Fig. S10. Quantification of the thickness of the reduced layers in $H_2$, $CH_4$ and $CH_2CHCH_3$. The figure shows the spectra at the Sn $M_5$ edge for the $SnO_2$ nanoparticles at the end of the treatment in $H_2$, $CH_4$ and $CH_2CHCH_3$. The fractions shown in the figure are obtained by dividing the area of the peak at ca. 489 eV (highlighted in grey), due to Sn(II) only by the area of the peaks at ca. 492.5 and 494.5 eV, both due almost only to Sn(IV) (highlighted in orange). If these fractions are called $f$, then $1\text{-}f = e^{-t/\lambda}$, where $\lambda$ is the electron escape depth, taken equal to 3.5 nm, gives the thickness $t$.

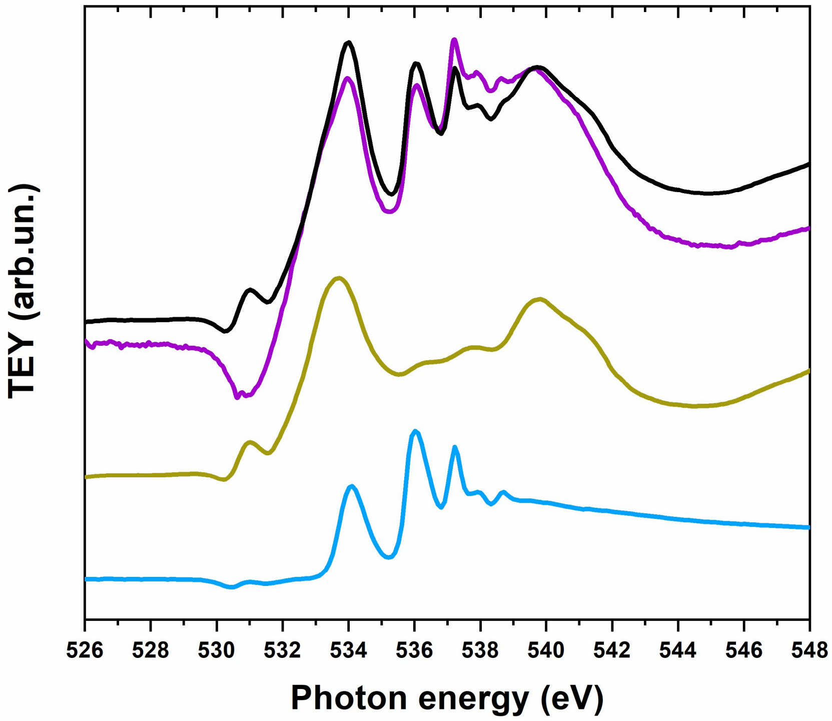


Fig. S11. Fitting of the O K-edge spectrum at 200 °C in flowing $H_2$ (black line) with a linear combination of the spectra of water in the gas phase and of the $SnO_2$ nanoparticles in vacuum. The fit is shown as a dark pink line. The spectra of water and of the nanoparticles weighted for the coefficients of the linear combination (0.29 and 0.71, respectively) are shown as dark cyan and dark yellow lines.

| **Energy (eV)** | **SnO** | **$SnO_2$** |
|---|---|---|
| **$M_5$** | | |
| 489.4 | + | - |
| 492.9 | + | + |
| 494.9 | + | + |
| 497.3 | + | + |
| **$M_4$** | | |
| 501.2 | + | + |
| 503.1 | + | + |
| 505.2 | - | + |

**Tab. S1.** Assignment of the different peaks at the Sn $M_{4,5}$-edges. +: appearing; -: non appearing.

(S1) Stöhr, J. *NEXAFS Spectroscopy*; Springer-Verlag Berlin Heidelberg, 1992.
